# Supplementary material for: Evolution of gene knockout strains of E. coli reveal regulatory architectures governed by metabolism
Source: Nat Commun. 2018 Sep 18;9:3796. doi: 10.1038/s41467-018-06219-9 (PMC6143558; doi:10.1038/s41467-018-06219-9)
Supplement: Supplementary file 1 — Supplementary Information [file 41467_2018_6219_MOESM1_ESM.pdf]

Supplementary Information

Laboratory evolution of gene knockout strains of *E. coli* reveal regulatory architectures that are governed by metabolism

McCloskey et al.

Supplementary Figure 1

Innovative transcript levels vs. Fitness change. Innovative transcripts are those transcript profiles (Ref - uKO - eKO, see Fig. 3) that classify as Novel, Overcompensated, Reinforced, or Unrestored (p-value < 0.05). Fitness change is calculated as the average distance in % fitness change from Ref to uKO, and from uKO to eKO. Average counts for transcript levels and average % fitness change are shown as y- and x- axis, respectively. Pearson correlation coefficient and p-value are annotated on the plot.

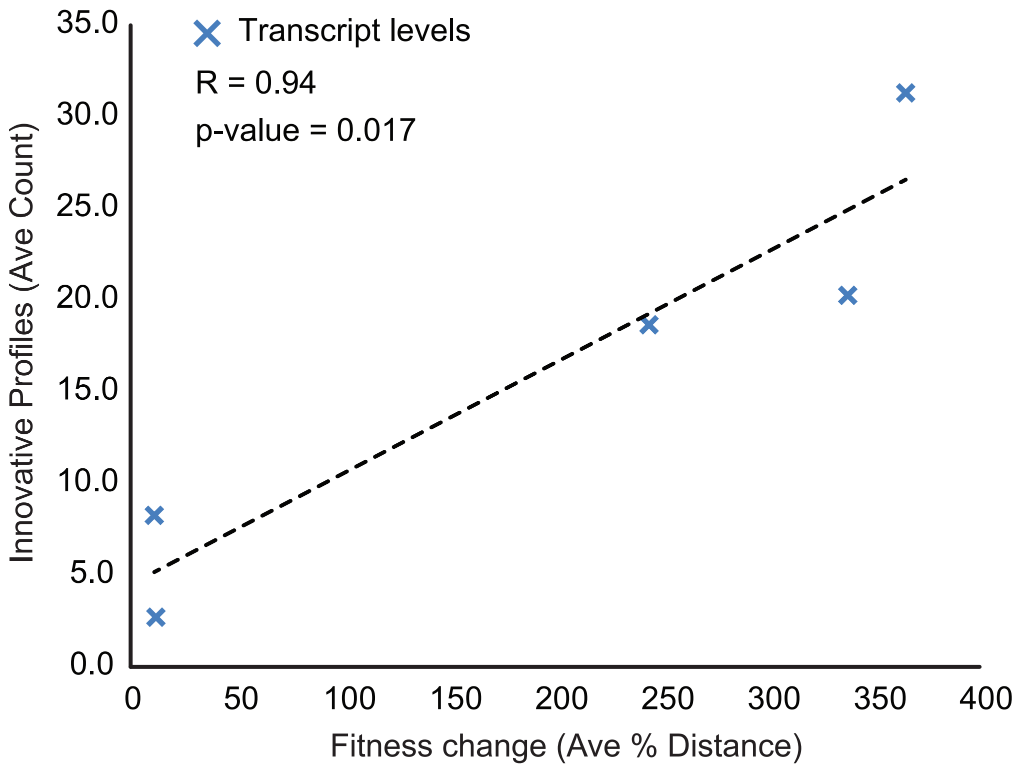

Transcript levels
R = 0.94
p-value = 0.017
Innovative Profiles (Ave Count)
35.0
30.0
25.0
20.0
15.0
10.0
5.0
0.0
0
50
100
150
200
250
300
350
400
Fitness change (Ave % Distance)
