# Supplementary material for: Evolution of gene knockout strains of E. coli reveal regulatory architectures governed by metabolism
Source: Nat Commun. 2018 Sep 18;9:3796. doi: 10.1038/s41467-018-06219-9 (PMC6143558; doi:10.1038/s41467-018-06219-9)
Supplement: Supplementary file 3 — Description of Additional Supplementary Files [file 41467_2018_6219_MOESM3_ESM.pdf]

Description of Additional Supplementary Files

File Name: Supplementary Data 1
Description: Reaction knockout selection in the reference strain. See Methods for details. The reaction name, mean and standard deviation for the sampled flux distributions, the number of genes, the reactants and products of the reaction that could be measured by the metabolomics assay used in the study, whether the gene was essential or expressed, whether a single knockout could remove the reaction, whether the reaction was coupled to any other reactions, notes on the target reaction deletion, and whether the reaction deletion was implemented are shown.

File Name: Supplementary Data 2
Description: Competing layers of regulation and discrepancies in regulatory annotation statistics. The descriptive statistic for competing layers of regulation per lineage or per regulatory are given. The descriptive statistics for discrepancies in regulatory annotation per lineage or per regulatory are given. Statistics are expressed based on counts within each lineage or percentages within each lineage.

File Name: Supplementary Data 3
Description: System component pairwise agreement and disagreements according to annotated biochemical pathways and regulatory networks. See Methods for details. The percent agreement and percent disagreement between component profiles and annotated regulation are given for each lineage. Parent classes not defined by EcoCyc include the following. GPR: regulatory relation between a gene, gene product (i.e., protein), and the reaction catalyzed by the gene product. Protein-Ligand-DNA-Binding-Reactions: regulatory relation between a small molecule, transcription factor (TF), and target gene. DNA-to-Protein-DNA-Binding-Reactions: regulatory relation between the expression of a TF, the TF, and target gene. RNA-to-Protein: regulatory relation between the expression of a TF and the consensus activation profile of the TF.

File Name: Supplementary Data 4
Description: Competing layers of regulation. See Methods for details. Consensus profiles of regulatory activity are given for individual regulator to regulated entity interaction (i.e., “consensus per name”) and combined regulator to all regulated entity interactions (i.e., “consensus per regulator”) for each lineage, perturbation, and across all perturbation. Consensus profiles of regulatory activity that were constructed using

only regulated entities with a single annotated transcription factor (TF) (i.e., “1 regulator”) are also given. A greater “weight” given to the consensus profile indicates a greater confidence.

File Name: Supplementary Data 5
Description: Annotated mutations. Table headers include the following (from left to right): 1) The type of mutation. Mutations include amplification (AMP), deletion (DEL), insertions (INS), mobile element aided insertions or deletions (MOB), single nucleotide polymorphism (SNP). 2) The frequency of the mutation in the end point lineage population. 3) The genes affected by the mutations. Mutations located in an intergenic region between two genes are shown with both genes separated by a semi-colon. 4) The annotation for the mutation. 5) The starting position of the mutation on the chromosome. 5) The name of the end-point lineage. 6) The location of the mutation. Locations include coding regions, regions associated with cryptic prophages, intergenic regions, regions two coding genes not classified as an intergenic region (intergenic/intergenic), and repetitive elements (REP or RIP). 7) The chromosome number of the mutation. In this case, 1 for all strains because *E. coli* has only one chromosome.

File Name: Supplementary Data 6
Description: Mutation statistics.
